# Finding a Needle in a Haystack: Identification of EGFP Tagged Neurons during Calcium Imaging by Means of Two-Photon Spectral Separation

**DOI:** 10.3389/fnmol.2012.00096

**Published:** 2012-10-29

**Authors:** Marco Brondi, Sebastian Sulis Sato, Luigi Federico Rossi, Silvia Ferrara, Gian Michele Ratto

**Affiliations:** ^1^National Enterprise for Nanoscience and Nanotechnology, Istituto Nanoscience, Consiglio Nazionale delle RicerchePisa, Italy; ^2^Institute of Neuroscience, Consiglio Nazionale delle RicerchePisa, Italy; ^3^Scuola Normale SuperiorePisa, Italy; ^4^Center for Nanotechnology Innovation@NEST, Istituto Italiano di TecnologiaPisa, Italy

**Keywords:** *in vivo* targeted two-photon microscopy, scattering, spectroscopy, oregon green bapta1, *in vivo* functional imaging, excitation spectra

## Abstract

The combination of two-photon *in vivo* imaging and genetic labeling of specific cell types in the mouse brain is a powerful method to refine our understanding of brain circuitry and to dissect the contribution of specific neural classes to cortical function. The synthetic calcium indicators are the best fluorescent reporters for cellular activity that are presently available but their spectral proprieties are often overlapped with those of the fluorescent proteins used for genetic labeling. Such is the case of Oregon Green BAPTA1 and EGFP, the most widely used fluorophores for targeted two-photon imaging. The emission spectra of these molecules are virtually identical, precluding their separation by narrow band filters at the detector side. However, even if their one photon excitation spectra are very similar, their two-photon excitation spectra differ significantly: here we show how it is possible to exploit this difference to separate the relative contributions of EGFP and Oregon Green to the total fluorescence signal. This approach addresses two different issues: the unbiased detection of cells expressing EGFP in a cortical volume injected with Oregon Green, and the computation of the Ca^2+^ insensitive fluorescence background. The latter data is essential for the quantitative comparison of the relative changes in Ca^2+^ concentration between different cells, containing variable concentrations of EGFP. This strategy can be easily extended to any couple of fluorophores provided that have a different two-photon excitation spectra.

## Introduction

In the last 30 years, imaging has become an essential tool to visualize changes in intracellular Calcium concentration in living cells (Zipfel et al., 2003). In neurons, the relevance of Ca^2+^ homeostasis is not only related to its role as second messenger, but, because of the activation of voltage sensitive channels, Ca^2+^ changes also reflect neuronal activity. Thus, Ca^2+^ imaging is a powerful tool to study coordinated neuronal activity (Grienberger and Konnerth, 2012). Furthermore, Ca^2+^ imaging of astrocytes is contributing to understand the complexity of their interaction with neurons and their role in shaping cortical excitability and synaptic plasticity (Jourdain et al., 2007; Halassa and Haydon, 2010; Navarrete and Araque, 2010; Poskanzer and Yuste, 2011; Takata et al., 2011). With the introduction of two-photon imaging, the capacity of imaging cell activation has been brought to bear to the intact brain, allowing the determination of the cooperative behavior of brain cells by analyzing simultaneously tens to hundreds of cells with single cell resolution and on temporal scales varying from 1 Hz to 1 kH (Grewe et al., 2010; Dal Maschio et al., 2012; Katona et al., 2012). As the analysis of brain circuitry is refined, the need arises to identify specific neuronal classes to assess their differential contribution to brain activity. In special cases, cells can be identified by means of their localization, by morphological criteria, or by their capacity of loading specific intravital dyes (Nimmerjahn et al., 2004; Langer and Helmchen, 2012). However, the only general tool is the use of a genetically encoded fluorescent tag expressed under a cell specific promoter (Feng et al., 2000; Tamamaki et al., 2003).

The most commonly used tag is the green fluorescent protein (EGFP), as there is a wide availability of vectors and of transgenic mice leading to cell specific EGFP expression. Unfortunately, the emission spectra of EGFP is overlapped on the emission spectra of the most useful calcium indicators, fluo-3 and Oregon green Bapta 1, and therefore these signals cannot be separated on the detection channels (Minta et al., 1989; Orte et al., 2005; Drobizhev et al., 2011).

Although OG, fluo-3, and EGFP share a very similar one photon excitation spectra, their spectral properties for two-photon excitation are quite different (Albota et al., 1998; Drobizhev et al., 2011). Here we show a strategy to allow the separation of EGFP and OG fluorescence by exploiting their differential two-photon excitation properties.

## Results and Discussion

**Figure 1:**
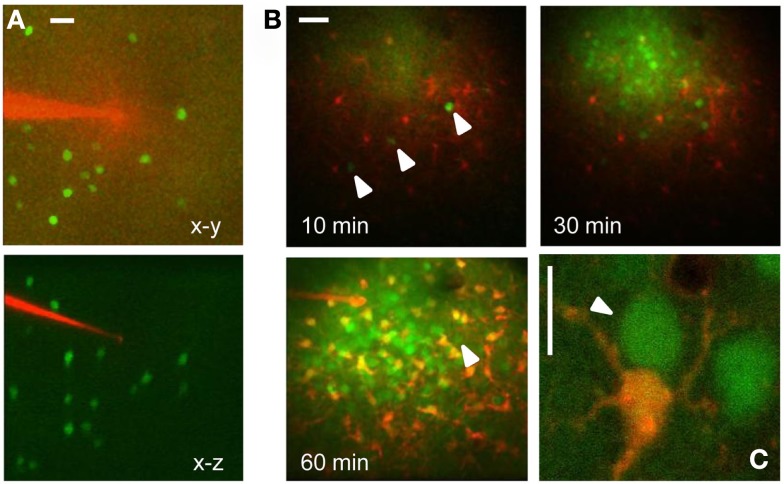
***In vivo* imaging of EGFP tagged neurons in the visual cortex**. **(A)** Low magnification stack of a cortical column just before microinjection of OG. The micropipette is loaded with the astrocyte dye SulfoRhodamine 101 and the images have been obtained with 820 nm excitation. The lower panel shows the transverse reconstruction of the column that is about 300 μm deep. Calibration bar 30 μm. **(B)** Time lapse sequence started 10 min after the end of the OG-SR101 injection. The three arrowheads show three neurons positive for EGFP. After 60 min from the beginning of loading the GAD67-EGFP neurons are no more distinguishable because of the OG fluorescence. The arrowhead indicates one of the three positive cells. Calibration bar 40 μm. **(C)** High magnification view of an astrocyte (red) and two neurons loaded with OG (excitation 820 nm). The arrowhead points to a GAD67-GFP neuron that has been identified before of the injection. Bar 10 μm.

**Figure 2:**
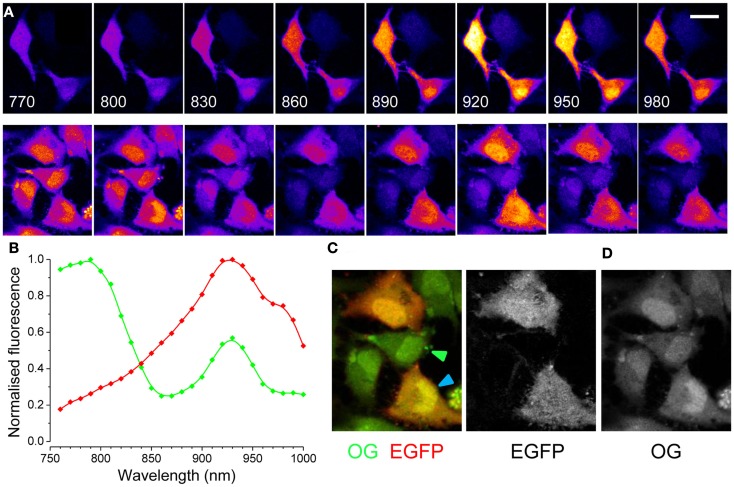
**Two-photon excitation spectra of EGFP and OG in cultured cells. (A)** Upper panels: the excitation spectra of EGFP is visualized in HeLa cells excited at various wavelengths. The excitation power was regulated in order to maintain a constant photon flux at the objective output at all wavelengths. Calibration bar 30 μm. Lower panels: cells transfected with EGFP have been also loaded with OG and the images show the resulting spectra. **(B)** Excitation spectra of EGFP (red, *n* = 4) and of OG (green, *n* = 3). The spectra of OG did not change with Ca^2+^ concentration, if not for a linear scaling (not shown). **(C)** Synthetic RGB image obtained by presenting the image excited at 790 nm in the green channel and the image excited at 930 nm in the red channel. **(D)** Spectral linear decomposition has been used to separate the contribution to fluorescence of EGFP and OG.

The targeted expression of EGFP allows the identification of specific cell types *in vivo*. As an example, Figure 1 shows the tagging of Parvalbumin-positive interneurons in the cortex *in vivo*. Here, EGFP is expressed under the control of the Gad67 promoter (Tamamaki et al., 2003), resulting in the staining of a sparse population of neurons, as shown in Figure 1A. EGFP positive neuron can be easily identified before the injection and loading of the Ca^2+^ dye Oregon Green Bapta 1 AM (OG). As the dye is hydrolyzed and becomes fluorescent, the EGFP positive neurons are not distinguishable any more since the emission spectra of EGFP and OG are virtually over imposed (Figures 1B,C). However, since there are indications that their two-photon excitation spectra differ substantially, we thought to exploit this difference to separate the two signals by proper selection of the excitation wavelength. We transfected cultured HeLa cells with a plasmid for EGFP (pEGFP-C1, Clontech) and we imaged them at the two-photon microscope. By tuning the wavelength of the pulsed laser and its power in order to maintain a constant photon flux at the objective output, we obtained the sequences depicted in Figure 2A. In the upper panel cells were carrying EGFP only, while the cells shown in the lower panels were loaded with OG and some also carried EGFP. From these sequences and the associated movies (see Movies S1 and S2 in Supplementary Material) it results that, while EGFP has a single excitation peak around 930 nm, OG has two peaks, one at 790 nm, where EGFP is only weakly fluorescent, and a secondary peak at 930 nm. The excitation spectra for OG1 and EGFP are shown in Figure 2B. All imaging data in this and following figures have been corrected by subtracting the PMTs mean background noise and offset.

We can exploit this information to solve two different problems: (a) the comparison of images obtained at the two peaks allows to discriminate cells carrying both fluorophores from those only loaded with OG or EGFP. (b) when computing the rate of Ca^2+^ changes, it is necessary to correct the OG signal for any contamination from EGFP, and this require to compute the contribution of EGFP at the wavelength used to excite the Ca^2+^ dependent signal. This is especially important with cells carrying a very high concentration of EGFP compared to OG.

A quick way of identifying cells carrying EGFP in a crowded field is presented in Figure 2C. This RGB image is obtained by combining two images collected at two different wavelengths: the red channel is the image excited at the peak for EGFP (930 nm), while the green channel has been collected at 790 nm, where the OG fluorescence prevails. Cells expressing EGFP appear red-orange, while non-transfected cells are green (see arrowheads). A more quantitative approach allows to separate the two signals: the spectra show that the secondary peak of OG (at 930 nm) is 1.75 times lower than the primary peak (at 790 nm). Therefore, we compute a new image defined as:

(1) $$I_{EGFP}=I\left(930\right)-\frac{I\left(790\right)}{1.75}$$

where *I*(930) and *I*(790) represent images obtained at excitations of 930 and 790 nm. The resulting image is mostly formed by EGFP fluorescence. In turn, this image can be subtracted from the image obtained at 930 nm to get the OG signal (Figures 2C,D). Both methods allow a quick identification of EGFP positive cells and can be easily implemented to screen for EGFP tagged cells.

These procedures needs only two images collected at the two excitation peaks. A more quantitative analysis relies on more images and can be implemented on a full spectroscopic scan, where images are collected at a series of wavelengths. The fluorescence *F(*λ*)*, due to the combination of OG and EGFP fluorescence, can be described by a linear combination of their spectra:

(2) $$F\left(\lambda_{i}\right)=\alpha \cdot OG\left(\lambda_{i}\right)+\beta \cdot EGFP\left(\lambda_{i}\right)$$

**Figure 3:**
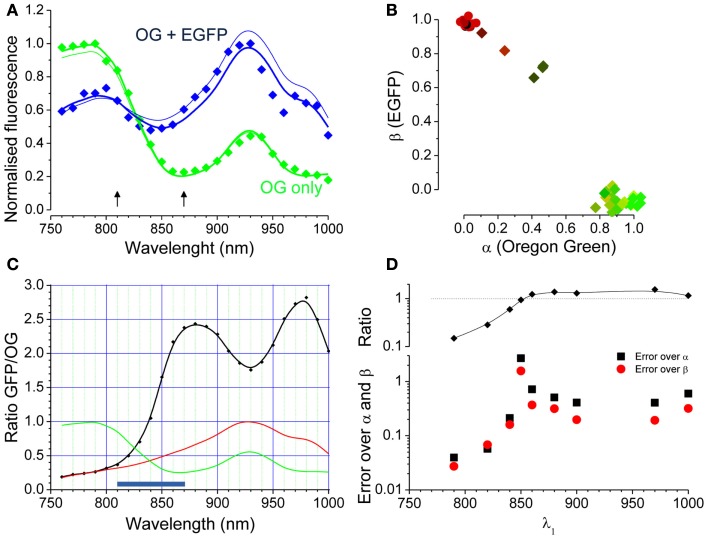
**Linear decomposition of the excitation spectra when both EGFP and OG are present**. **(A)** The dots are the measured spectra of the two cells indicated by arrowheads in Figure 2. The thick lines show the linear composition of the OG and EGFP spectra with α and β computed by fitting the full spectral series. The thin lines are the spectral reconstructions computed from only two images obtained at 810 and 870 nm (arrows). **(B)** The scatter plot of the α and β parameters shows that data points populate the plot according with the spectral signature of each cell. **(C)** The black curve reports the ratio between the EGFP and OG reference spectra (red and green curves respectively). **(D)** α and β have been computed using images obtained at two wavelengths and the plot represents the difference between this estimate and the estimate obtained by fitting of the full spectra. One wavelength was fixed at 930 nm and the second varied as plotted in the graph. The average differences between the estimates (black and red symbols for α and β respectively) were computed by applying this procedure on the data obtained from 31 cells fluorescent for OG and EGFP in various degrees. The upper panel shows the ratio [EGFP(λ_1_)/OG(λ_1_)]/[EGFP(λ_2_)/OG(λ_2_); with λ_2_ = 930 nm]. As expected, the two wavelength estimates get worse when the ratio is close to 1.

Where the two unknown parameters α and β describe the relative contributions of the two fluorophores. If two images obtained at two different wavelengths are available, Eq. 2 becomes a system of two linear equations with two unknowns (α and β, see later). If imaging data obtained at several excitation wavelengths are available, like in the spectral series of Figure 2A, the system is over determined and can be solved numerically. Figure 3A shows the spectral data for the two cells indicated by the arrowheads of corresponding colors in Figure 2C. The continuous lines derives from fitting the data with the linear combination of the OG and EGFP spectra shown in Figure 3B. The relative contribution of EGFP and OG to the observed fluorescence is quantified by the values of α and β. The cell with mixed contribution has α = 0.51 and β = 0.69, while the second cell has only OG fluorescence (α = 1.03 and β = −0.09). We measured the excitation spectra of 45 cells from two different set of experiments. The first group of cells was transfected with EGFP only, while the second group was also loaded with OG 2 days after the transfection. The spectra were obtained from the imaging sequences and the terms α and β were computed. The scatter plot of Figure 3B shows that cells can be easily classified in terms of these two parameters. Cells stained with either EGFP or OG are tightly clustered at the extreme ends of the plot, while cells with mixed spectra fall in between.

Although the complete spectral series measure the relative spectra contribution with good accuracy, it is quite cumbersome to obtain. Of course, α and β can be determined by a far smaller number of observation. In fact, as we already mentioned, α and β can be computed by the direct solution of the system provided that two images are obtained at two different wavelength:

(3) $$\left\{\;\begin{array}{c} F\left(\lambda_{1}\right)=\alpha \cdot OG\left(\lambda_{1}\right)+\beta \cdot GFP\left(\lambda_{1}\right)\\ F\left(\lambda_{2}\right)=\alpha \cdot OG\left(\lambda_{2}\right)+\beta \cdot GFP\left(\lambda_{2}\right) \end{array}\right.$$

The solution of this linear system is given by:

$$\begin{array}{llll} & \alpha =\frac{F\left(\lambda_{1}\right)-\beta \cdot GFP\left(\lambda_{1}\right)}{OG\left(\lambda_{1}\right)} & & \text{(4)}\\ & \beta =\frac{OG\left(\lambda_{1}\right)\cdot F\left(\lambda_{2}\right)-OG\left(\lambda_{2}\right)\cdot F\left(\lambda_{1}\right)}{OG\left(\lambda_{1}\right)\cdot GFP\left(\lambda_{2}\right)-OG\left(\lambda_{2}\right)\cdot GFP\left(\lambda_{1}\right)} & & \text{(5)} \end{array}$$

The choice of λ_1_ and λ_2_ is important because it determines the reliability of the estimates for α and β. The two wavelengths must be chosen in order to maximize the difference between:

(6) $$\frac{GFP\left(\lambda_{1}\right)}{OG\left(\lambda_{1}\right)}\text{ and }\frac{GFP\left(\lambda_{2}\right)}{OG\left(\lambda_{2}\right)}$$

If for a couple (λ_1_, λ_2_) the two terms above are similar, the Eq. 3 are almost dependent and the solution is poorly determined. Figure 3C shows that, since the EGFP/OG ratio is not a monotonic function of λ, there are couples of wavelengths that provide very poor estimates of α and β, while other couples work much better. To illustrate this fact, we have computed the error of fitting the spectra by observing only two wavelengths by comparing the values of α and β obtained by Eqs 4 and 5 with the values obtained by fitting the entire spectra. The average errors have been computed on 31 cells keeping λ_2_ fixed at 930 nm while varying λ_1_. This data are plotted in Figure 3D showing a great variability of the error depending on the condition expressed by Eq. 6. These data assist in determining what wavelengths should be used to estimate α and β. At first glance fixing the two points at 790 and 970 nm offers the largest change in ratio, but there are two disadvantages: at 790 the fluorescence of EGFP is very weak and interference from autofluorescence that might arise *in vivo* is a concern. Also, the difference in wavelengths is very conspicuous (180 nm), leading to potential problems when λ-dependent scattering and absorption occur (see later; Blanca and Saloma, 1998; Oheim et al., 2001). Furthermore, it is helpful to select wavelengths at which the OG and EGFP fluorescence is not too small in order to allow reliable measurements. All things considered, the couple 810 and 870 nm represents a better choice: this interval encompasses the region of largest slope of the EGFP/OG ratio and, although far away from the excitation maxima for both spectra, offers a reasonable signal at both wavelengths. Finally, the wavelength difference is quite small (60 nm) which helps in reducing the problems associated with absorption and scattering of excitation. The thin lines in Figure 3A show the computed spectra obtained from only two images acquired with an excitation of 810 and 870 nm.

**Figure 4:**
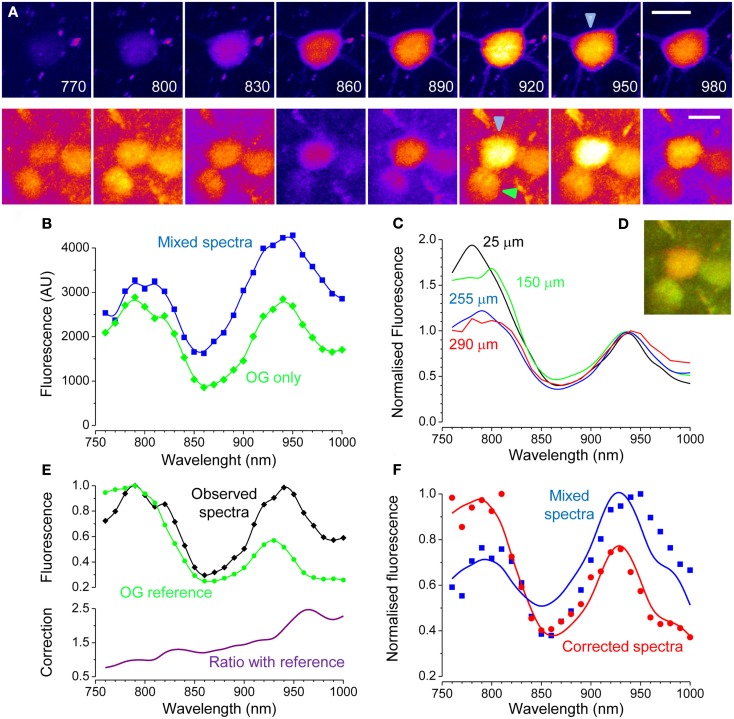
**Excitation spectra of EGFP and OG *in vivo***. **(A)** Imaging was performed on a transgenic mouse expressing EGFP in a sparse subset of cortical neurons. The upper panels shows a spectral series of an EGFP expressing neuron. The lower panels show the same field after loading with OG. The focal plane lies about 150 μm underneath the cortical surface. Calibration bars 10 μm. **(B)** Spectra measured after loading of two different cells. Green: OG measured in an EGFP negative neuron. Blue: mixed spectra measured in the neuron of the upper panel. **(C)** OG spectra measured *in vivo* at different imaging depths (56 cells from two mice): the spectra distortion caused by scattering and absorption increases with increasing imaging depth. All spectra have been normalized to the value of the rightmost peak. **(D)** RGB composition of the images acquired with 820 and 920 excitation. The expression of EGFP is deduced by the orange-red color of the cell body. **(E)** Comparison of the OG spectra (black) recorded from one neuron (green arrowhead in panel **(A)** with the reference spectra (green). The observed spectra is obviously distorted, with a strong loss of fluorescence at shorter excitation wavelengths. The lower panel shows this effect by plotting the ratio between the distorted spectra and the reference spectra. **(F)** The ratio with the reference spectra has been used to correct the spectra from the EGFP cell loaded with OG (original data: blue symbols, corrected data: green symbols). The original spectra cannot be fitted properly by the linear decomposition (α = 0.53; β = 0.71; blue line), that, in contrast, is very accurate after correction (α = 0.92; β = 0.26; red line).

Next, we performed similar experiments by employing a transgenic mouse line expressing EGFP under the promoter Thy 1.1 (line M of Feng et al., 2000). The spectra of a EGFP positive cell body placed at about 150 μm of depth is shown in Figure 4A. After micro injection of OG, the cell bodies of two adjacent neurons became visible. The green trace in Figure 4B shows the OG excitation spectra of the neuron indicated by the green arrowhead, while in blue is plotted the EGFP–OG combined spectra of the central neuron (blue arrowhead). One very interesting fact emerges upon inspection of the OG spectra: although it exhibits two peaks as in culture, its shape is obviously distorted. Indeed, the peaks of OG, that have quite different amplitude in Figure 2B, now are almost identical. We supposed that the shape of the spectra was distorted because of scattering of the infrared excitation in the brain tissue. If this were true, one would expect that the entity of this effect depends on the imaging depth. Indeed, Figure 4C shows how the peak at 790 nm of OG became less and less prominent as the imaging depth increased. It should be noted that we found a great variability between different animals and at different locations in the same animal: this was likely due to the varying degree of superficial vascularisation of the cortex. Scattering increases at shorter wavelengths, with a power law that depends on the characteristics of the scattering media (Oheim et al., 2001). Given the non-linearity of two-photon microscopy, even if the available excitation would decrease proportionally to the wavelength, the resulting fluorescence would decrease with the square of the wavelength (Helmchen and Denk, 2005). Therefore, even if the excitation is so regulated to provide a constant photon flux at the objective output, less and less power is available within the tissue at shorter wavelength. This might put into question the excitation wavelength used to image OG: the usual choice is 820 nm which is close to the *in vitro* peak of excitation (790 nm) and also allows the simultaneous excitation of rhodamine. From these data it could be argued that in thick samples, 920–940 nm could be a wiser choice, depending on the actual scattering occurring in the specific preparation. The evaluation of scattering and absorption is important when computing the offset in the OG signal caused by EGFP, but it does not much influence the qualitative separation of EGFP expressing cells that we showed in Figures 2C and 4D shows that the EGFP positive cell is clearly recognizable.

The decomposition of the mixed spectra described by Eq. 2 requires the knowledge of the OG and EGFP spectra that are best obtained *in vitro*. However, this cannot be done unless the spectra distortion caused by scattering and absorption of the tissue is compensated. We reasoned that since the shape of the OG spectra is independent on Ca^2+^ concentration, we can estimate the change in available power at the focal plane by comparing the spectra measured *in vivo* to the reference spectra obtained in absence of scattering and absorption. This is done by computing the ratio between the observed spectra and the OG reference spectra and this corrective factor can be used to adjust the shape of the mixed OG-EGFP spectra. In general it is always possible to do this, since the EGFP tagging is meaningful only if a minority of cells are tagged. Even in absence of clearly resolved cell bodies, the spectra of OG can be obtained from the fluorescence of the neuropile. This correction is described by the following equation:

(7) MS(λ)=MSObs(λ)OGRef(λ)OGObs(λ)

Where *MS_Obs_* is the observed spectra for the cells with mixed OG and EGFP fluorescence; *OG_Obs_* is the observed spectra of cells with OG fluorescence only; *OG_Ref_* is the OG spectra measured in absence of scattering and absorption, and MS is the corrected spectra of the mixed fluorescence. This procedure is illustrated in Figures 4E,F. Interestingly, the efficacy of this procedure can be tested by comparing the result of the linear decomposition of the observed and corrected spectra as shown in Figure 4F.

Although Ca^2+^ imaging is not quantitative, it is important to be able to compare the amplitude of the relative Ca^2+^ changes between different cells (Grewe et al., 2010). For this estimate the absolute fluorescence is not relevant, since it depends on the actual concentration of the indicator in the imaged cells, and it is more appropriate to compare the relative changes in fluorescence. The presence of EGFP in some cells contaminates the OG functional measure, introducing a background signal that is not Ca^2+^ sensitive. Thus the interpretation of the physiological activity may be compromised since the proportionality between the relative change of fluorescence and the amplitude of the Ca^2+^ transient is the critical assumption used to estimate electrical activity patterns and kinetics.

Around the K_d_ of the indicator (430 nM for Oregon Green Bapta 1) Ca^2+^ concentration changes linearly with the relative change of fluorescence defined as:

(8) $$\frac{\Delta F}{F}\left(t\right)=\frac{F\left(t\right)-F_{0}}{F_{0}-\text{background}}$$

**Figure 5:**
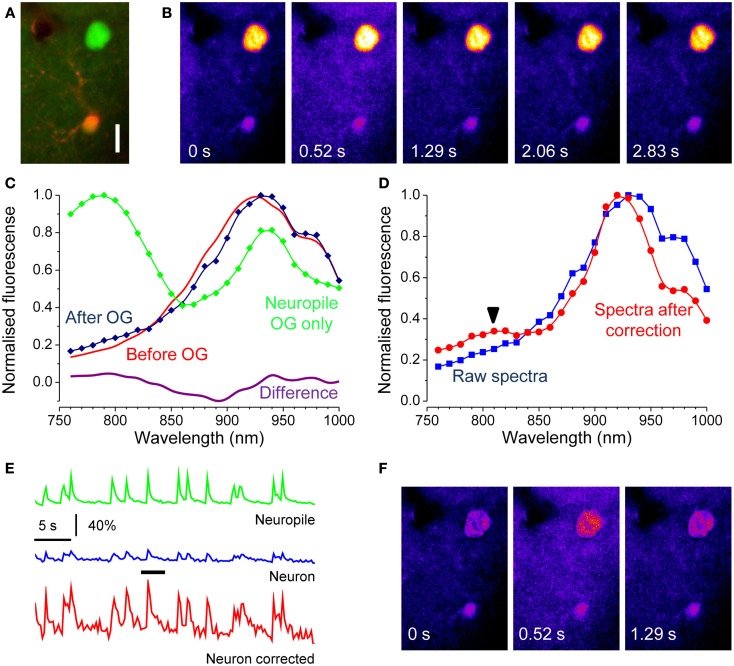
**Correction of the Ca^2+^ insensitive background during dynamic imaging**. **(A)** the field, imaged at a depth of about 100 μm from the cortical surface, contains a neuron strongly expressing GFP. After loading with OG, both the red astrocyte and the neuropile show a faint green fluorescence. The image has been stretched non-linearly to better visualize the neuropile fluorescence. Bar 10 μm. **(B)** Time series obtained from the raw data showing one episode, indicated by the black bar in panel E, of rhythmic epileptiform activity in the visual cortex. **(C)** Spectra of the EGFP expressing cell body before and after OG loading (red and blue lines respectively). Loading was very low to maximize the interference of EGFP with Ca^2+^ imaging. The magenta line in the lower panel shows the difference between these spectra showing a noisy but unmistakable signature of OG. The green trace shows the spectra of the neuropile surrounding the cell body. **(D)** Spectra of the neuron cell body before and after correction for scattering and absorption. The raw spectra does not allow to detect the OG signature by linear decomposition, in fact, the fit returns α = −0.05 and β = 1.00. After correction, the presence of the OG spectral signature becomes obvious and that is reflected in the values of the α and β parameters: α = 0.11; β = 0.80. **(E)** Time course of the Ca^2+^ changes occurring during epileptiform activity measured for the neuropile, that returns the average activity of the local circuit, and for the tagged neuron. The response computed from the raw data is very small, due to the strong Ca^2+^ insensitive offset. Equation 8 and 9 were used to correct for the offset, and the resulting Ca^2+^ oscillations are now comparable with the responses recorded in the neuropile (red trace). **(F)** The Ca^2+^ insensitive background can be subtracted from the imaging sequence to provide a correct representation of the Ca^2+^ dynamics. Here, the Ca^2+^ oscillations of the soma are much more visible.

Where *F(t)* is the OG fluorescence obtained from a time series and *F_0_* is the baseline level, usually obtained by averaging fluorescence during periods of no Ca^2+^ activity. The term *background* is the fraction of fluorescence that is not Ca^2+^ sensitive. If the cell expresses EGFP, this contribution must be obtained in order to estimate correctly the changes in intracellular Ca^2+^. Next, we demonstrate how this can be done. In a set of experiments we have imaged neurons at the border of the OG injection, where only a modest amount of indicator is loaded. In this situation, most of the fluorescence originates from EGFP and the correction of the Ca^2+^ insensitive signal is extremely important for a correct quantification of activity. This is illustrated in Figure 5A that shows a strongly fluorescent neuron in a field of faint OG fluorescence. Here, we triggered the onset of periodic Ca^2+^ oscillations by treating the surface of the cortex with the GABA_A_ inhibitor Bicuculline Methiodide (Gomez-Gonzalo et al., 2011). The time lapse sequence shows a single Ca^2+^ transient, and it is apparent that only a very little change in fluorescence can be detected in the cell soma (Movie S3 in Supplementary Material). We reasoned that the offset due to EGFP at any wavelength can be estimated by the linear decomposition of the excitation spectra. Figure 5C shows the spectra of the neuron before and after the OG loading (red and blue traces respectively): the differences in the spectra caused by loading is minute but, the subtraction between the two shows the characteristic bimodal spectra of OG (magenta). By measuring the fluorescence in the neuropile, we have also obtained a good spectra for OG: the relatively small difference between the amplitude of the two peaks of OG shows a strong spectra distortion caused by the tissue. By using the relationship 7, we corrected the excitation spectra of the neuron. As shown in Figure 5D, the correction for absorption and scattering returns a spectra with a small but unmistakable component of OG (arrow). Then, by linear decomposition of this spectra, we obtained the α and β terms. Now the intensity of EGFP fluorescence at the wavelength *λ_ex_* at which the dynamic Ca^2+^ imaging experiment is performed can be computed by:

(9) $$\text{backgroun}{\text{d}}_{ex}=F\left(\lambda_{ex}\right)\frac{\beta \cdot GFP_{\text{ref}}\left(\lambda_{ex}\right)}{\alpha \cdot OG_{\text{ref}}\left(\lambda_{ex}\right)+\beta \cdot GFP_{\text{ref}}\left(\lambda_{ex}\right)}$$

In the example shown in Figure 5, EGFP fluorescence is much stronger than that of OG and, in fact, about 79% of the signal imaged at 830 nm originates from the fluorescent protein. Thus, it is to be expected that the correction for the Ca^2+^ insensitive background would radically modify the amplitude of the response. Indeed, Figure 5 shows that the amplitude of the transient measured at the neuron soma is drastically affected. The correction can be extended to the entire time lapse sequence, allowing for a more representative view of the relative changes of Ca^2+^ in the cell body and dendrites (Figure 5F and Movie S4 in Supplementary Material).

Here we have characterized the spectral properties of two fluorophores *in vivo* and we have used these data to allow spectral un-mixing of fluorescence to identify cells tagged with EGFP and to correct Ca^2+^ dynamics. We have also shown a strong effect of imaging depth on the effective excitation available at the imaging plane. In principle, the quantitative approach we have described could be extended to others combinations of fluorophores provided that have sufficiently different two-photon excitation spectra. We envisage the possible development of a multi-color cell tagging scenario combined with Ca^2+^ dynamic measures.

## Materials and Methods

### Cell cultures

HeLa cells were cultured in Dulbecco Modified Eagle’s Medium (DMEM) supplemented with 1 mM Sodium Pyruvate, 2 mM l-glutamine, 10 U/mL–10 μgmL Penicillin-Streptomycin, 10% bovine fetal serum, 10 mM HEPES. Two days before the imaging session the cells were transfected with a pEGFP-C1 vector (Clontech) with Effectene Transfection Reagent (QUIAGEN).

### Animals and surgical procedures

All experimental procedures were conducted in accordance with the ethical guidelines of the Istituto Superiore di Sanità. Experiments were performed on C57Bl/6J, C57Bl/6J -*Thy1*::EGFP (M line), and C57Bl/6J – *Gad67*::EGFP 25 mice (males and females) between postnatal day 28 and 70. Mice were anesthetized with an intraperitoneal injection of Urethane (20% w/V in physiological saline, 20 mg/Kg Urethane). An intramuscular injection of dexamethasone sodium phosphate (2 mg/kg body weight) was administered to reduce the cortical stress response and cerebral edema during the surgery. During the experiment the animal respiration was aided providing O_2_ enriched air and body temperature monitored and held constant at 37°C with a feedback-controlled heating blanket (Harvard Instruments).

The animal head was shaved and 2.5% lidocaine gel applied to the scalp. Scissors were used to cut the flap of skin covering the skull of both hemispheres; the exposed bone was washed with saline and periosteum gently removed with a pair of forceps to provide a better base for glue and dental cement to adhere to.

The highest cortical EGFP expressing areas was identified with transcranial epifluoresce illumination. A custom-made steal head post with a central imaging chamber was then glued with cyanoacrylate in a plane approximately parallel with the skull over the cortical region of interest and cemented in place with white dental cement (Paladur).

The mouse was head-fixed and a craniotomy of 2–3 mm in diameter was drilled over the region of interest; care was taken to minimize heating of the cortex during surgery, dural tears, or bleeding, and to keep the cortex superfused with sterile ACSF(126 mM NaCl, 3 mM KCl, 1.2 mM KH_2_PO_4_, 1.3 mM MgSO_4_, 26 mM NaHCO_3_, 2.4 mM CaCl_2_, 15 mM Glucose, 1.2 mM HEPES in distilled H_2_O, pH 7.4). During functional calcium imaging experiments, stereotyped interictal activity was induced superfusing the cortex with Bicuculline Methiodide (BMI, Sigma, 2 mM in ACSF).

### Multi-cell bolus loading

The cortex was bulk loaded with a micro-injections of the calcium indicator dye Oregon Green Bapta-1AM (Molecular Probes, Invitrogen) and the selective astrocyte marker Sulforhodamine 101 (Molecular Probes, Invitrogen; Garaschuk and Konnerth, 2010).

OGB-1AM was first dissolved in 4.5 μL DMSO containing Pluronic F-127 (50 μg, Molecular Probes, Invitrogen) and further diluted 1/11 in 45 μL of ACSF, to a final concentration of 0.8 mM. SR101 (40 μM) was added to the solution to distinguish astrocytes from neurons. The staining solution was sonicated for 10 min at 37°C to assure complete solubilization of OGB-1AM, then filtered with 0.20 μm PVDF/Nylon filters and loaded into a borosilicate micropipette (Sutter Instruments, 1–2 μm tip diameter).

The micropipette was a inserted at an angle into the cortex to the desired depth (150–200 μm) with a micromanipulator (MP285, Sutter Instruments) and the dye was slowly pressure injected (0.5–0.7 bar, 2–3 min) under visual control (10 × air objective). 1–1.5 h were necessary to observe maximum stable fluorescent labeling of neurons and astrocytes.

### Two-photon *in vivo* spectroscopy and calcium imaging

Fluorescence was imaged with a Prairie Ultima Multiphoton microscope (Prairie technologies) and a mode-locked Ti:sapphire laser (Chameleon Ultra II, Coherent) through a 40 × (0.8 NA) water immersion objective (Olympus). Before each imaging session we measured the power of the excitation laser at the optic bench and at the output of the objective lens at each wavelength. Given this conversion function, the power at the sample, that is not accessible once the mouse is placed under the objective, could be computed from the power measured on the optic bench. The power was measured with a radiometer (Melles Griot). The conversion factor was calculated as:

(10) rλ=PwObjectivePwBench

During spectra acquisition, the excitation power was controlled by means of a Pockel Cell (Conoptics) in order to maintain a constant photon number impinging on the sample at all wavelengths:

(11) $$n_{\varphi }\alpha Pw.\lambda$$

The conversion factor *r*_λ_ was used to calculate the photon number on the sample at each power value read on the optic bench by using the relationship:

(12) $$n_{\varphi \text{sample}}\alpha Pw_{\text{Bench}}\cdot r_{\lambda }\cdot \lambda$$

Fluorescent cells were imaged at variable depth in the cortex. The average laser power delivered to the brain surface was 20 mW. PMT gain was kept constant at 667 V since previous calibrations showed that this voltage gives the best S/N ratio.

*Z*-series (vertical resolution 1 μm, 256 × 256 pixels) around the cell bodies of interests were acquired every 10 nm of wavelength from 760 nm to 1000 nm. To compensate for the focal plane drift due to chromatic aberrations, the *Z*-series was adjusted at each wavelength to optimize the optical slicing of the cell bodies of interest. During functional calcium imaging cortical activity was monitored by imaging fluorescence changes at 820 nm. *T*-series of fields of variable size were acquired at 2–5 Hz to detect activity-related somatic calcium signals.

At the end of the imaging session, a *Z*-series was acquired for *post hoc* evaluation of the depth of the imaged fields. A dark frame with the laser shutter closed was also acquired to measure the mean thermal noise arising in the PMTs and the pedestal usually added by the electronics.

### Image analysis

Twelve-bit image sequences were analyzed with ImageJ (NIH). *Z*-series were projected (maximum intensity) and subtracted of the dark background. For each spectrum, *Z*-projections were mounted in a sequence from 760 to 1000 nm and aligned for tangential drift with IRIS (http://www.astrosurf.com/buil/us/iris/iris.htm). Regions of Interest (ROIs) were manually drew around cellular somata: all pixels within each ROI were averaged to give the fluorescence variation as a function of the excitation wavelength. *T*-series from functional calcium imaging were subtracted of the dark background.

## Conflict of Interest Statement

The authors declare that the research was conducted in the absence of any commercial or financial relationships that could be construed as a potential conflict of interest.

## Supplementary Material

The Supplementary Material for this article can be found online at http://www.frontiersin.org/Molecular_Neuroscience/10.3389/fnmol.2012.00096/abstract
